# Constructing effective energy functions for protein structure prediction through broadening attraction-basin and reverse Monte Carlo sampling

**DOI:** 10.1186/s12859-019-2652-5

**Published:** 2019-03-29

**Authors:** Chao Wang, Yi Wei, Haicang Zhang, Lupeng Kong, Shiwei Sun, Wei-Mou Zheng, Dongbo Bu

**Affiliations:** 1 Key Lab of Intelligent Information Processing, Institute of Computing Technology, Chinese Academy of Sciences, 6, Kexueyuan South Road, Zhongguancun, Beijing, 100190 China; 2 University of Chinese Academy of Sciences, 19-1, Yuquan Road, Shijingshan, Beijing, 100049 China; 3 Institute of Theoretical Physics, Chinese Academy of Sciences, 55, Zhongguancun East Road, Beijing, 100190 China

**Keywords:** Protein structure prediction, Energy function, Attraction-basin, Reverse Monte Carlo sampling, Monte Carlo search, Linear program

## Abstract

**Background:**

The *ab initio* approaches to protein structure prediction usually employ the Monte Carlo technique to search the structural conformation that has the lowest energy. However, the widely-used energy functions are usually ineffective for conformation search. How to construct an effective energy function remains a challenging task.

**Results:**

Here, we present a framework to construct effective energy functions for protein structure prediction. Unlike existing energy functions only requiring the native structure to be the lowest one, we attempt to maximize the attraction-basin where the native structure lies in the energy landscape. The underlying rationale is that each energy function determines a specific energy landscape together with a native attraction-basin, and the larger the attraction-basin is, the more likely for the Monte Carlo search procedure to find the native structure. Following this rationale, we constructed effective energy functions as follows: *i*) To explore the native attraction-basin determined by a certain energy function, we performed reverse Monte Carlo sampling starting from the native structure, identifying the structural conformations on the edge of attraction-basin. *ii*) To broaden the native attraction-basin, we smoothened the edge points of attraction-basin through tuning weights of energy terms, thus acquiring an improved energy function. Our framework alternates the *broadening attraction-basin* and *reverse sampling* steps (thus called BARS) until the native attraction-basin is sufficiently large. We present extensive experimental results to show that using the BARS framework, the constructed energy functions could greatly facilitate protein structure prediction in improving the quality of predicted structures and speeding up conformation search.

**Conclusion:**

Using the BARS framework, we constructed effective energy functions for protein structure prediction, which could improve the quality of predicted structures and speed up conformation search as well.

**Electronic supplementary material:**

The online version of this article (10.1186/s12859-019-2652-5) contains supplementary material, which is available to authorized users.

## Background

Determination of protein structure is important for understanding protein functions [1]. The classical techniques for protein structure determination include X-ray crystallography, nuclear magnetic resonance, and electron microscopy. These determination techniques, however, often suffer from the limitations in both expensive costs and long determination period, leading to the ever-increasing gap between the number of known protein sequences and that of solved protein structures [2]. Computational approaches to protein structure prediction from sequences are becoming increasingly important to narrow down the gap [3].

The protein structure prediction approaches can be categorized into two families, namely, template-based modeling [4–10] and *ab initio* approaches [11–15]. Recently the predicted contacts have also been shown to be invaluable to protein structure prediction [16–21]. Unlike the template-based modeling approaches, the *ab initio* prediction approaches work without requirements of known similar protein structures. Briefly speaking, most *ab initio* prediction approaches are based on the hypothesis that the native structure of a protein should be the highly-populated one with sufficiently low energy; thus, *ab initio* approaches usually perform conformation search to find a structural conformation with sufficiently low energy. For example, Rosetta employs the Monte Carlo technique to search conformations assembled from fragments of known structures, and finally reports the centroid of a large cluster of low-energy conformations [11].

For the *ab initio* prediction approaches, one of the key issues is designing an effective energy function [11, 14, 15]. Typically, an energy function is a weighted-sum of multiple energy terms. The energy terms characterize specific structural features, especially the interplay between local and global interactions of residues. For example, the hydrophobic interaction term was designed to capture the observed tendency of non-polar residues to aggregate in aqueous solution and exclude water molecules. Van der Waals force term is the sum of the attractive or repulsive forces among residues. Hydrogen bonding term describes the electromagnetic attractive interaction between polar molecules in which hydrogen is bound to highly electronegative atom oxygen in the carboxyl [1]. In Rosetta, a total of 13 energy terms were used at the residue level, and over 140 terms were used at the full-atom level; therefore, it is important to find the optimal weighting of so many energy terms [11]. This study focuses on designing an optimal weighting of the 13 energy terms used in Rosetta.

Ideally, an effective energy function is expected to be able to distinguish the native structure from non-native conformations (called *decoys*), and could drive as much as possible initial conformations to the native-like one during the conformation search process. To achieve these two objectives, a widely-used strategy for designing energy function is to maximize the correlation between energy and quality of decoys [22]. Here the quality of a decoy refers to the structural similarity between the decoy with the native structure, which is measured using root mean square deviation (RMSD) of backbone atoms in this study. Inspired by the idea of “funnel-shaped free energy surface”, Levitt et al. proposed a funnel sculpting technique to construct energy functions that allow the conformation search procedure to easily “roll” into the native structure from a random starting conformation [23]. In another study, Shell et al. attempted to smooth energy function to make the energy landscape a funnel [24].

In this study, we present a framework that constructs effective energy function for protein structure prediction. Our framework, called BARS, consists of two procedures, i.e., *broadening attraction-basin* where the native structure lies in the energy landscape (hereafter denoted as *native attraction-basin*), and *reverse sampling*. The underlying rationale is that the larger the attraction-basin is, the more likely for the Monte Carlo procedure to find the native structure. To explore the attraction basin, we performed reverse sampling starting from the native structure. Subsequently, we tuned the weights of energy terms to broaden the native attraction-basin and thus acquired an improved energy function. We showed that both the possibility of successful search and the quality of predicted conformation increase when using the improved energy function.

The manuscript is organized as follows: “Methods” section describes the whole framework of our method, and the linear program model to optimize protein energy weights as well. “Results and discussion” section lists experimental results of the optimized energy function. In “Conclusion” section, we will discuss some limitations of our method and possible future works.

## Methods

To construct an effective energy function, our BARS framework alternates two procedures, i.e., for an energy function, we first explored the native attraction-basin in the corresponding energy landscape using reverse sampling, and then improved the energy function through broadening the attraction-basin. These two procedures were alternated until the energy function changes sufficiently small between successive iterations as below.


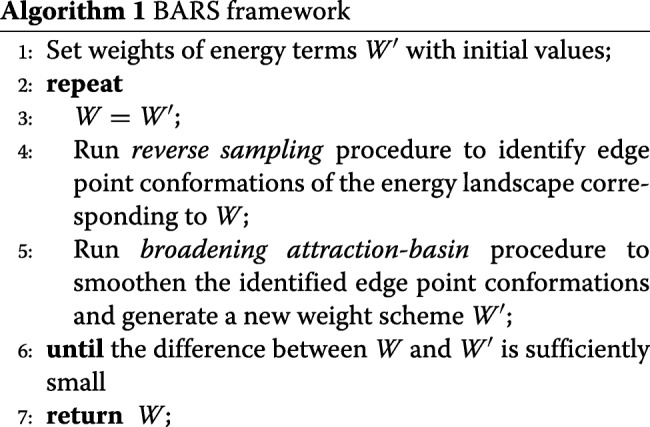


The details of the two procedures are described as follows.

### Exploring the native attraction-basin using *reverse sampling*

When applying Monte Carlo technique to search the structural conformation with the lowest energy, the *ab initio* approaches might finally end with success if starting from some initial structural conformations, and might end with failure if starting from other initial structural conformations. An initial structural conformation is said to lie in the native attraction-basin if the conformation can finally evolve into the native structure during the conformation search process.

**Fig. 1:**
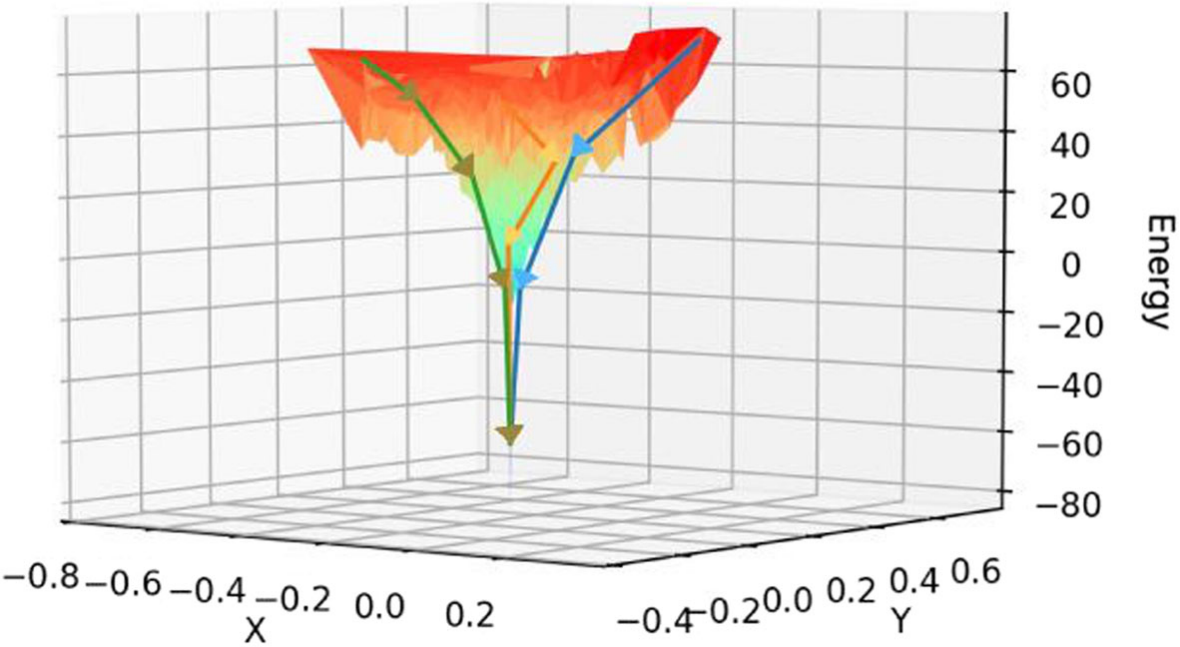
Conformation searching process using the Monte Carlo technique. The protein structure prediction approaches usually employ the Monte Carlo technique to search the conformation with the lowest energy. An execution of conformation search will generate a path of conformations, e.g., the lines in blue and yellow. Here the energy landscape was drawn using 1000 decoys of protein 1ctfA: we calculated the RMSD among all possible pairs of decoys, and then performed principal component analysis of the generated RMSD matrix [23]. The *x* and *y* axises represent the first and second principal components, respectively. Decoy energy was calculated using *score*3 of Rosetta

To explore the native attraction-basin under a specific energy function, we propose a technique called *reverse Monte Carlo sampling*. Here, the term “reverse” comes from the fact that the sampling process works essentially reverse to the general Monte Carlo technique used for conformation search. Specifically, the general Monte Carlo search procedure starts from a random initial conformation and moves towards the native structure, during which the energy of conformation is reducing. For each inter-mediate structural conformation, a perturbation is made to generate a new conformation. Some popular perturbation techniques include fragment replacing used by Rosetta [11] and torsion angle sampling used by FALCON [13]. The newly-generated conformation is accepted if it has lower energy relative to the original conformation; otherwise the new conformation will be rejected with a probability according to the Metropolis-Hasting rule [25] (Fig. 1). To emphasize the difference between the general Monte Carlo search technique and the sampling technique used in this study, we denoted the former one as *forward Monte Carlo* technique.

**Fig. 2:**
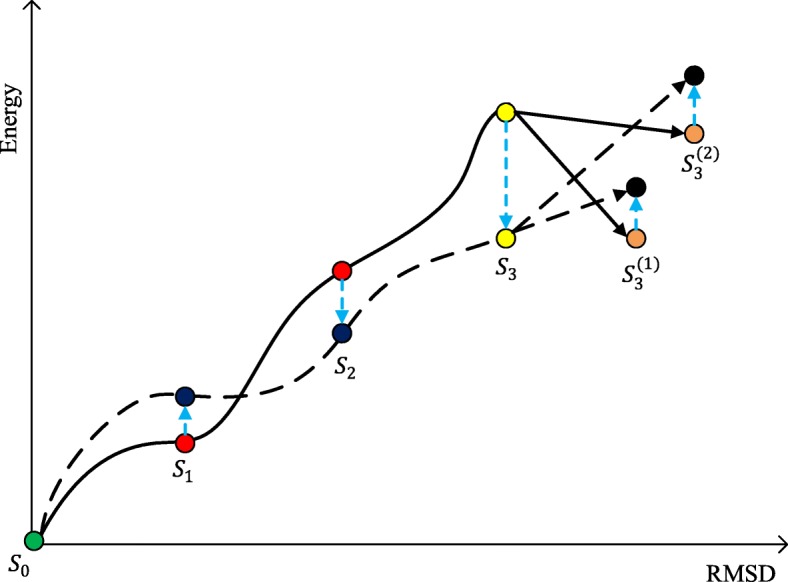
Reverse Monte Carlo sampling and tuning weights of energy terms. Reverse Monte Carlo sampling starts from the native structure and moves towards the edge of the native attraction-basin, during which the energy of conformations is increasing. Here the solid line shows a path of conformations generated by reverse Monte Carlo sampling *S*_0_→*S*_1_→*S*_2_→*S*_3_. *S*_3_ represents an edge point conformation as its perturbation neighbors, e.g., $S_{3}^{1}$ and $S_{3}^{2}$, have lower energy than *S*_3_. The dash line shows these conformations after tuning weights of energy terms, where *S*_3_ is no longer the edge point conformation as one of its perturbation neighbors, $S_{3}^{2}$ has larger energy than *S*_3_. Note that during weight-tuning, the monotonicity of energy is maintained within the native attraction-basin

On the contrary to the forward Monte Carlo search technique, our reverse Monte Carlo sampling procedure starts from the native structure and moves towards the edge of the native attraction-basin, during which the energy of conformations is increasing (Fig. 2). The reverse sampling process ends at a conformation if any perturbation of this conformation cannot leads to increase of energy. Intuitively, this conformation lies at the edge of the native attraction-basin and thus is denoted as *edge point conformation* in this study.

Formally, each execution of reverse sampling will generate a path of conformations *P*=*S*_0_→*S*_1_→*S*_2_→...→*S*_*n*_, where *S*_0_ denotes the native structure, *S*_*i*_(1≤*i*≤*n*) denotes the *i*th inter-mediate conformations along the reverse sampling path, and *S*_*n*_ denotes the final edge point conformation. The energy of the inter-mediation conformations increases along the path, suggesting the monotonicity of energy within the native attraction-basin. The RMSD between *S*_0_ and *S*_*n*_ is calculated as a rough measure of the radius of native attraction-basin. For the edge point conformation *S*_*n*_, we perform *m* times of perturbation, thus acquiring *m* perturbation neighbors of *S*_*n*_, denoted as $N(S_{n})=\left \{S_{n}^{(1)}, S_{n}^{(2)},..., S_{n}^{(m)}\right \}$ (Fig. 2). In our study, *m* is set as 1000.

### Broadening the native attraction-basin by smoothening the edge point conformations

Intuitively, if we can “smoothen” the edge point conformations, the native attraction-basin will be broadened since the reverse sampling process will not be stuck at these edge point conformations. We accomplished the “smoothening” operation through tuning weights of energy terms such that the energy of *S*_*n*_ is decreased to be less than at least one of its perturbation neighbors $S_{n}^{(i)} (1 \le i \le m)$. To make the native structure still the lowest one under the new energy function, we imposed a constraint on weight-tuning such that after tuning weights, the new energy of *S*_*i*_ should be lower than that of the conformation *S*_*i*+1_. In other words, the monotonicity of energy are maintained within the attraction-basin, and thus the shape of the native attraction-basin will also be maintained. Figure 2 shows how the conformation path changes after improving energy function.

We designed a linear program to calculate the optimal weights that satisfy this constraint.

1 $$ \begin{aligned} &\text{min}\qquad ||W-W_{0}|| \\ s.t. W \cdot {E}_{i} &\leq W\cdot {E}_{i+1}, 0\leq i\leq n-1 \end{aligned}  $$

2 $$ W \cdot {E}_{n} \leq \frac{1}{m}\sum\limits_{j=1}^{m} W\cdot {E}_{n}^{(j)}  $$

3 $$ W \geq 0  $$

4 $$ |W| = |{W}_{0}|  $$

Here the vector *W* denotes the weights of energy terms, and *W*_0_ denotes the original weights before tuning. For an inter-mediate conformation *S*_*i*_ in the reverse sampling path, *E*_*i*_ denotes the vector of its energy terms, i.e. $E_{i}= < e_{i}^{(1)},e_{i}^{(2)},\ldots,e_{i}^{(13)} >$, where $e_{i}^{(k)}$ represents the *k*-th energy term. The objective of the linear program is to find a new weighting scheme with change as small as possible. Formula (1) describes a constraint that the original relative order of *S*_*i*_ and *S*_*i*+1_, i.e., the monotonicity of energy within the native attraction-basin, should be kept after tuning weights. Formula (2) was designed to “smoothen” the edge point conformation, i.e. at least one of the *m* perturbation neighbors of the edge point conformation has a higher energy; thus, *S*_*n*_ is no longer an edge point conformation under the new energy function.

## Results and discussion

### Data set

We evaluated the BARS framework on Test101 dataset that contains a total of 101 benchmark proteins. The criteria for selecting these proteins are: *i*) The length of these proteins are less than 120 amino acids as the energy terms used in this study were designed for small proteins [11]. *ii*) These proteins cover most SCOP superfamilies as energy functions differ with protein class. We used three proteins as representatives of three SCOP classes (Table 1) to explain the working process of the BARS framework and summarized experimental results on Test101 dataset in Additional file 1: Table S1.

**Table 1:** Three benchmark proteins used in the study

PDB ID	Chain	Class	#Residues	# *α* helices	# *β* strands
1ctf	A	*α* + *β*	68	4(38)	3(18)
1ilo	A	*α*/ *β*	77	3(27)	4(18)
1iie	A	all *α*	75	3(42)	-

The 3 proteins come from 3 different SCOP classes: all *α* (Class A), *α*/ *β* (Class C), *α* + *β* (class D). Residue numbers are 68, 77, 75, respectively. Columns 5 and 6 shows the number and total length of *α* helices and *β* strands

### Analysis of evolution of energy functions

As mentioned above, the BARS framework alternates the *broadening attraction-basin* and *reverse sampling* steps to gradually improve energy function. In our experiment, the initial weights of energy terms were set as the weights used by Rosetta in the scoring function *score*3.

Table 2 shows how the energy functions evolve as iteration proceeds for protein 1ctfA. From this table, it can be observed that the weights of energy terms are almost fixed after 6 iterations. In addition, although the difference between consecutive iterations are not very large as expected, the final weights are quite different from the initial ones (Manhattan distance: 15.16).

**Table 2:** Weights of energy terms during the iteration process of BARS

		Weights at iteration					
Energy terms	Initial weights	#1	#2	#3	#4	#5	#6
Env	1.00	1.00	0.97	0.48	0.49	1.26	1.30
Pair	1.00	1.23	1.22	0.64	2.71	2.62	2.59
Vdw	1.00	1.06	0.88	0.61	0.55	0.55	0.55
Hs	1.00	1.70	1.70	1.06	0.09	0.09	0.09
Ss	1.00	1.00	1.00	0.48	0.48	0.48	0.48
Sheet	1.00	1.00	1.00	1.00	3.48	3.48	3.48
R-sigma	1.00	1.00	1.00	0.53	0.53	0.53	0.53
Cb	1.00	0.02	0.01	0.41	0.00	0.00	0.05
Rg	3.00	3.00	3.14	6.05	0.49	0.49	0.49
Contact order	1.00	1.00	1.05	0.74	0.00	0.00	0.00
Ramachandran	0.00	0.00	0.00	0.01	0.91	1.01	1.02
Hb-srbb	0.00	0.00	0.02	0.00	3.27	2.49	2.41
Hb-lrbb	0.00	0.00	0.00	0.00	0.00	0.00	0.00

Here, the initial weights were set as the weights used by Rosetta in *score*3. A total of 6 iterations are shown here. The Manhattan distances of each adjacent weighting are 1.97, 0.44, 6.62, 17.47, 1.74, 0.21, respectively. The iteration process stopped when the Manhattan distance is less than a threshold of 0.3. The cosine of angle of weight vectors at iterations *#*4 and *#*5 is 0.98, while that of iterations *#*5 and *#*6 reaches 0.99

We also compared the final weights for proteins in different classes. As shown in Table 3, the final weights exhibited considerable difference for proteins from different classes. For example, the weight of Env term in 1iieA (class A) is 5.69, about 4 times larger than that of 1ctfA (class D), and over 2.5 times larger than that of 1iloA (class C). This is consistent with the fact that the environment local geometrical term is more important for all- *α* proteins, since local residue-residue interactions dominate the helix formation process. In addition, 1ctfA (class D) can be distinguished from the other two proteins at the sheet term: the final weight of this term is 3.48 for 1ctfA, much larger than that of 1iloA (1.90) and 1iieA (1.14). This is also reasonable as *α* + *β* proteins usually contain anti-parallel *β*-sheets, whereas *α*/ *β* proteins contains *β*- *α*- *β* motifs. Taken together, the table supports the view point that different energy terms are emphasized for proteins in different classes.

**Table 3:** The optimized weights of energy terms calculated by BARS for protein 1ctfA (after 6 iterations), 1iloA (after 5 iterations), 1iieA (after 5 iterations)

Energy terms	Initial weights	Final weights	Final weights	Final weights
		for 1ctfA	for 1iloA	for 1iieA
Env	1.00	1.30	1.98	5.69
Pair	1.00	2.59	2.02	1.09
Vdw	1.00	0.55	0.72	0.20
Hs	1.00	0.09	0.82	1.28
Ss	1.00	0.48	0.37	0.20
Sheet	1.00	3.48	1.90	1.14
R-sigma	1.00	0.53	1.11	0.19
Cb	1.00	0.05	0.24	1.62
Rg	3.00	0.49	2.63	0.31
Co	1.00	0.00	0.00	0.62
Ramachandran	0.00	1.02	0.43	0.27
Hb-srbb	0.00	2.41	0.80	0.36
Hb-lrbb	0.00	0.00	0.00	0.00

The Manhattan distance of the weights of 1ctfA and 1iieA is 15.56, and that between 1ctfA and 1iloA is 12.39, while that between 1iloA and 1iieA is only 8.95. The cosine values of weight vectors are 0.63, 0.47, 0.77, respectively

### Broadening attraction-basin as iteration proceeds

We further investigated whether the native attraction-basin could be broadened after smoothening the edge point conformations. To measure the size of the native attraction-basin, we performed reverse sampling for 50 times, thus acquiring 50 edge point conformations. The mean RMSD between these edge point conformations and the native structure is calculated and used to measure the size of the native attraction-basin. Intuitively the calculated mean RMSD can be treated as radius of the native attraction-basin.

**Fig. 3:**
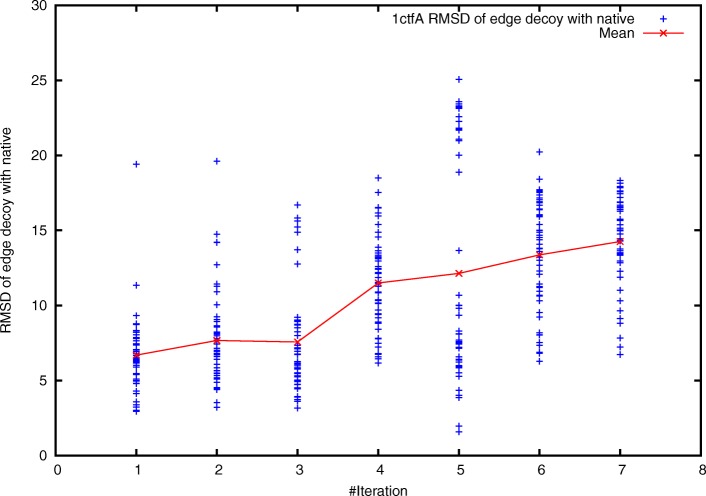
Broadening the native attraction-basin of protein 1ctfA as iteration proceeds. Here, *x*-axis denotes the number of iterations, and *y*-axis denotes the RMSD between the native structure and the edge point conformations. For the weighting scheme at each iteration, a total of 50 paths were generated, thus yielding 50 edge point conformations. The mean RMSD increases as iteration proceeds, suggesting that the native attraction-basin was broadened

As shown in Fig. 3, the mean RMSD was 6 Å initially, and increased to nearly 14 Å at the final iteration. This clearly suggested that the attraction basin was really significantly enlarged as iteration proceeds.

### Protein structure prediction using the improved energy function

We further investigated whether the improved energy function could facilitate protein structure prediction or not. For this aim, we compared the predicted structures by running Rosetta with different energy functions. Specifically, for each iteration step of BARS, we used the corresponding weighting scheme of energy terms to construct an energy function, and then run Rosetta using this energy function to generate 1000 decoys. We investigated two aspects of these predicted decoys: *i*)*Quality of final prediction results:* Among these decoys, a clustering procedure was executed and the centroid of the largest cluster was reported as the final prediction. Then we analyzed RMSD of the final prediction with the native structure. *ii*)*Good decoy ratio:* Among these generated decoys, we calculated the ratio of “good” decoys. Here, we adopted the widely-used criterion that for small proteins, a decoy is called *good decoy* if its RMSD to the native structure is less than 6 Å [13].

**Fig. 4:**
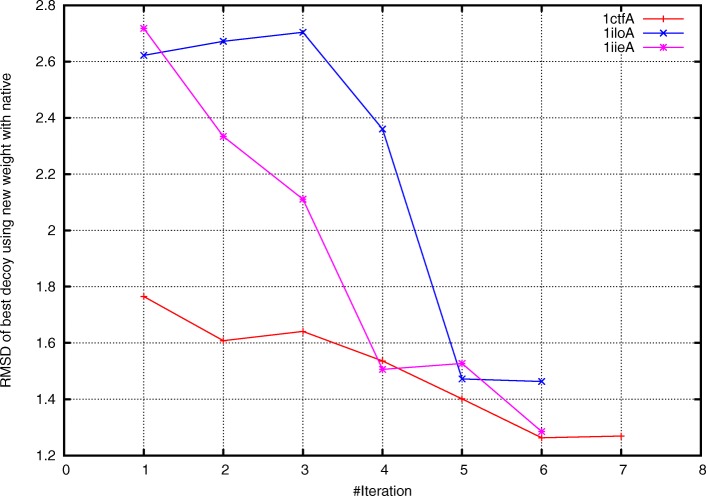
Quality of the final prediction for protein 1ctfA, 1iloA, and 1iieA. At each iteration step, a total of 1000 decoys were generated by Rosetta with corresponding weights of energy terms. We run clustering procedure for the 1000 decoys and finally selected the centroid of the largest cluster as the best decoy. The RMSD of the best decoys reduces as iteration proceeds, suggesting that the quality of prediction results increases

**Fig. 5:**
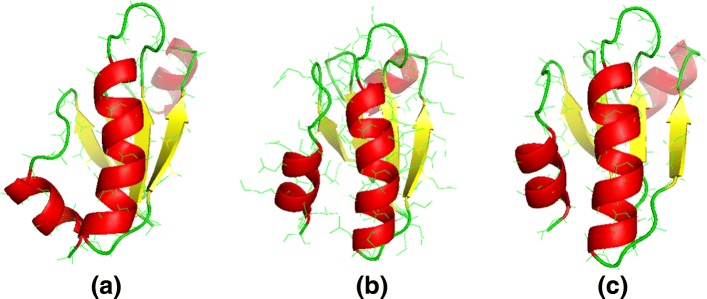
Predicted structures for protein 1iloA. Left panel: the predicted structures using the initial weights (RMSD: 2.7 Å). Middle panel: native structure. Right panel: the predicted structures using the optimized weights (RMSD: 1.3 Å). Thus, the optimized weights help improve the quality of predicted structures

**Fig. 6:**
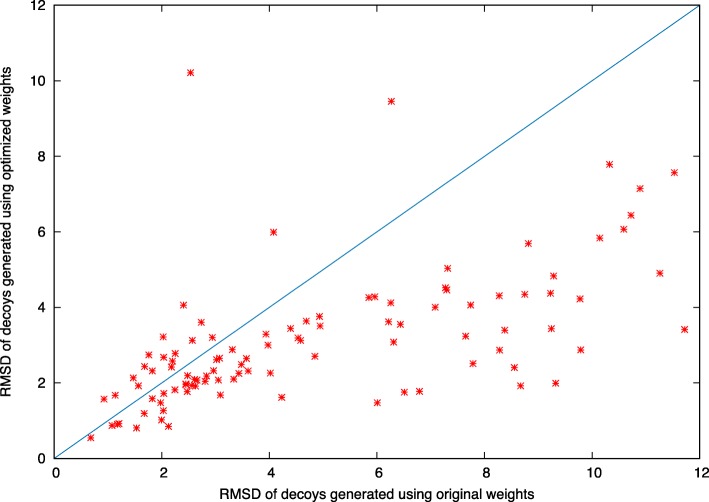
Quality of predicted structures using the original weights and the optimized weights of energy terms on 101 benchmark proteins of the Test101 dataset. For each benchmark protein, we predicted structures using both original weights and optimized weights of energy terms, and showed RMSD of the predicted structures as *x*-axis and *y*-axis, respectively. Most proteins fall below the diagonal line, suggesting that when using the optimized weights of energy terms, the predicted structures usually have smaller RMSD

As shown in Fig. 4, the quality of the final prediction results improved as iteration proceeded. Taking protein 1iloA as an example, the final prediction had a RMSD of 2.7 Å when using the original weighting scheme (Fig. 5, left panel). In contrast, when using the optimized weighting scheme, the quality of the final prediction structure improved (RMSD: 1.3 Å, Fig. 5, right panel). We repeated this experiment on the 101 benchmark proteins in Test101 set and observed that for 82 proteins, the quality of final prediction structure improved (Fig. 6 and Additional file 1: Table S1). For example, the quality of predicted structure for protein 1pchA was low (RMSD: 11.721 Å) when using the original energy weight; in contrast, when using the optimized weighting scheme, the predicted structure significantly improved (RMSD: 3.417 Å).

**Fig. 7:**
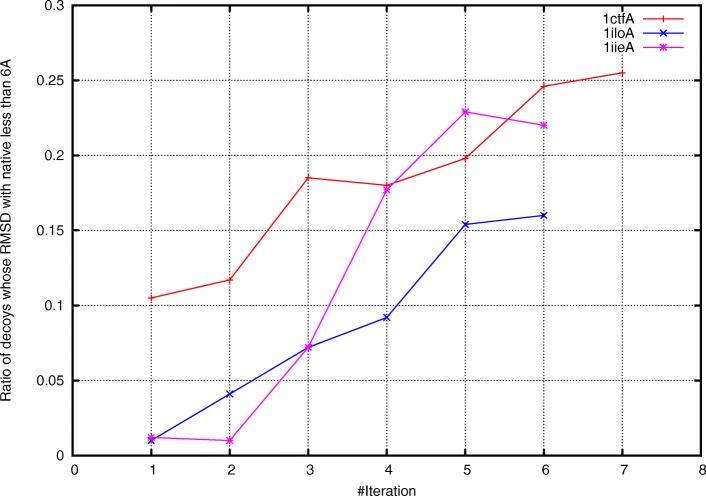
Good decoy ratio increases as iteration proceeds. At each iteration step, a total of 1000 decoys were generated by Rosetta using corresponding weights of energy terms. Here a decoy is called “good decoy" if it has a RMSD less than 6 Å to the native structure. The figure suggests that the “good decoy ratio" significantly increases, e.g. the ratio increases from 0.01 to over 0.2 for protein 1iloA. Thus, Rosetta can generate high-quality decoys more efficiently

Besides the quality of the final prediction, the good decoy ratio also increased considerably (Fig. 7). For example, if using the initial weights for protein 1iloA, only 11 decoys among the 1000 decoys were good decoys. In contrast, over 200 good decoys were generated when using the optimized weighting scheme. Taken together, these results clearly suggest that using the energy function constructed with BARS, the general Monte Carlo search procedure significantly improved in both success possibility and the quality of prediction results. This improvement should also be attributed to the broadened native attraction-basin.

### Application range of the constructed energy functions

Application range is one of the key issues of energy functions. An ideal energy function is expected to be applicable on a large amount of proteins rather than a single protein. To examine the application range of the energy functions acquired using BARS, we run Rosetta with the energy function acquired from protein 1ctfA on other seven benchmark proteins in the same class to 1ctfA. As shown in Table 4, on all of the seven proteins, the predicted structures have high quality (RMSD less than 7 Å). More importantly, on five out of the seven proteins, the prediction results using the energy function acquired from 1ctfA are much better than those predicted using the original weights.

**Table 4:** Quality of predicted structures using the original weights of energy terms and the weights acquired from protein 1ctfA

		Quality of the predicted structure	
Protein	SCOP family	Using original weights	Using weights acquired from 1ctfA
1osdA	d.58.17.1	3.221 Å	2.053 Å
1cpzA	d.58.17.1	2.810 Å	2.054 Å
1eigA	d.9.1.1	1.786 Å	2.071 Å
4ubpA	d.8.1.1	7.723 Å	3.286 Å
1ekzA	d.50.1.1	4.411 Å	3.448 Å
1dtjA	d.51.1.1	3.726 Å	6.249 Å
1ulrA	d.58.10.1	10.322 Å	6.948 Å

On all of the seven benchmark proteins, the predicted structures have RMSD less than 7 Å. On five out of the seven benchmark proteins, the predicted structures using the weights acquired from 1ctfA are better than those predicted using the original weights

Similarly, we run Rosetta with the energy function acquired from protein 1iieA on other 20 benchmark proteins. As shown in Table 5, on 19 out of the 20 benchmark proteins, the predicted structures have RMSD less than 5 Å. On 18 out of these benchmark proteins, the predicted structures using the weights acquired from 1iieA are better than those predicted using the original weights.

**Table 5:** Quality of predicted structures using the original weights of energy terms and the weights acquired from protein 1iieA

		Quality of the predicted structure	
Protein	SCOP family	Using original weights	Using weights acquired from 1iieA
1p7iA	a.4.1.1	0.688 Å	0.549 Å
1am9A	a.38.1.1	2.129 Å	1.074 Å
1cktA	a.21.1.1	1.675 Å	1.147 Å
1oqpA	a.39.1.5	1.697 Å	1.273 Å
1n1jB	a.22.1.3	2.029 Å	1.567 Å
1nkpA	a.38.1.1	1.991 Å	1.645 Å
1nkpB	a.38.1.1	2.444 Å	1.685 Å
1bw5A	a.4.1.1	2.273 Å	1.891 Å
1of9A	a.64.1.4	1.945 Å	2.053 Å
1q08A	a.6.1.3	2.584 Å	2.316 Å
1dgnA	a.77.1.3	10.376 Å	2.577 Å
1ow5A	a.60.1.2	3.082 Å	2.659 Å
1aoyA	a.4.5.3	3.035 Å	2.756 Å
1hstA	a.4.5.13	3.987 Å	3.031 Å
3ygsP	a.77.1.3	8.804 Å	3.14 Å
1psrA	a.39.1.2	4.700 Å	3.451 Å
1pueE	a.4.5.21	9.321 Å	3.545 Å
1ngrA	a.77.1.2	8.480 Å	3.942 Å
1hb6A	a.11.1.1	4.138 Å	4.542 Å
1ctjA	a.3.1.1	9.693 Å	6.29 Å

On 19 out of the 20 benchmark proteins, the predicted structures have RMSD less than 5 Å. On 18 out of these benchmark proteins, the predicted structures using the weights acquired from 1iieA are better than those predicted using the original weights

In summary, these results demonstrate the wide application range of the energy functions acquired using the BARS framework. A reasonable explanation of this wide application range is that proteins in a class might share similar folding process; thus, the energy function optimized on a certain protein is also applicable for other proteins in the same class.

## Conclusion

In this study we report the BARS framework for constructing effective energy functions. The framework attempts to improve energy function gradually such that the native attraction-basin was broadened. During this process, a reverse Monte Carlo sampling strategy was proposed to explore the native attraction-basin. Extensive experimental results demonstrate both effectiveness and wide application range of the constructed energy functions.

It has been reported that protein folding is a hierarchical process. According to this observation, Rosetta employs a multi-step prediction strategy. In particular, Rosetta first uses score function *score*_0_ with only hydrophobic core terms, then uses *score*_2_/*score*_5_ with secondary structure terms, and finally uses *score*_3_ to incorporate a total of 13 energy terms [11]. This study focuses on the optimization of weights for the third step. How to design effective energy functions for the first and second steps remains as one of our future works. We also noticed that on 19 out of the 101 benchmark proteins, the quality of prediction results using the optimized weighting scheme were low. How to design better energy functions for these proteins is another future work. It should be pointed out that the RMSD deviation of the 50 edge point conformations is large at iteration 5 for protein 1ctfA (Fig. 3). This might imply the irregular shape of the native attraction-basin. We will investigate this issue in future work.

The application of the BARS framework is not limited to protein structure prediction. Constructing an effective scoring function is usually the first important step for optimization problems in various domains such as RNA structure prediction, natural language processing, etc. How to optimally combine multiple terms into a scoring function is a challenging task. Our BARS framework should greatly facilitate designing effective scoring functions for a large variety of problems.

## Additional file

Additional file 1 Quality of predicted structures using the original weights and the optimized weights of energy terms on 101 benchmark proteins. (PDF 82 kb)

## Abbreviations

BARS: Broadening attraction-basin and reverse sampling
RMSD: Root mean square deviation
