# Supplementary material for: Constructing effective energy functions for protein structure prediction through broadening attraction-basin and reverse Monte Carlo sampling
Source: BMC Bioinformatics. 2019 Mar 29;20(Suppl 3):135. doi: 10.1186/s12859-019-2652-5 (PMC6439974; doi:10.1186/s12859-019-2652-5)
Supplement: Supplementary file 1 — Quality of predicted structures using the original weights and the optimized weights of energy terms on 101 benchmark proteins. (PDF 82 kb) [file 12859_2019_2652_MOESM1_ESM.pdf]

Supplementary Information

Constructing effective energy functions for protein structure prediction through broadening attraction-basin and reverse Monte Carlo sampling

Chao Wang[1,2†], Yi Wei[1,2†], Haicang Zhang[1,2], Lupeng Kong[1,2], Shiwei Sun[1,2], Wei-Mou Zheng[3,2^] and Dongbo Bu[1,2*^]

[1]Institute of Theoretical Physics, Chinese Academy of Sciences

[2]University of Chinese Academy of Sciences

[3]Institute of Theoretical Physics, Chinese Academy of Sciences

Supplementary Tables:

Table S1. Quality of predicted structures using the original weights and the optimized weights of energy terms on 101 benchmark proteins of the **Test101** dataset.

Protein	SCOP family	RMSD (Using original weights)	RMSD (Using optimized weights)
1a0aA	a.38.1.1	2.25	2.779
1am9A	a.38.1.1	2.126	0.849
1aoyA	a.4.5.3	3.019	2.614
1arkA	b.34.2.1	7.083	4.004
1b0xA	a.60.1.2	1.531	0.806
1b34A	b.38.1.1	9.324	1.993
1b3aA	d.9.1.1	6.007	1.474
1bdoA	b.84.1.1	10.891	7.144
1bo9A	a.65.1.1	1.205	0.921
1bw5A	a.4.1.1	2.245	1.817
1bxyA	d.59.1.1	4.231	1.617
1c7vA	a.39.1.5	2.18	2.42
1cc8A	d.58.17.1	2.474	1.772
1cf7A	a.4.5.17	3.606	2.317
1cktA	a.21.1.1	1.135	1.675
1cpzA	d.58.17.1	2.806	2.041
1ctjA	a.3.1.1	6.268	9.454
1cxyA	d.120.1.1	8.749	4.346
1d3bA	b.38.1.1	3.091	1.68
1dgnA	a.77.1.3	2.536	10.212
1dokA	d.9.1.1	8.671	1.921
1dtjA	d.51.1.1	6.216	3.618
1dxsA	a.60.1.2	1.997	1.019
1eigA	d.9.1.1	6.792	1.777
1ekzA	d.50.1.1	4.395	3.44
1el0A	d.9.1.1	7.787	2.509
1fseA	a.4.6.2	1.177	0.906
1fxrA	d.58.1.4	4.019	2.261
1g1cA	b.1.1.4	11.529	7.569
1g2tA	d.9.1.1	3.339	2.101
1gccA	d.10.1.2	2.568	3.126
1gvdA	a.4.1.3	2.629	1.918
1h3lA	a.177.1.1	1.472	2.133
1hb6A	a.11.1.1	4.079	5.99
1hstA	a.4.5.13	3.976	3.002
1htaA	a.22.1.2	2.031	1.265

1ichA	a.77.1.2	7.742	4.064
1ig4A	d.10.1.3	7.315	5.034
1ip9A	d.15.2.2	9.285	4.831
1irzA	a.4.1.11	3.435	2.251
1jo0A	d.68.4.1	6.312	3.082
1jo8A	b.34.2.1	6.258	4.12
1joyA	a.30.2.1	2.94	3.204
1k4uS	b.34.2.1	7.274	4.518
1k5nB	b.1.1.2	9.227	4.374
1k61A	a.4.1.1	1.077	0.87
1k8bA	d.241.1.1	2.036	2.681
1k99A	a.21.1.1	3.938	3.292
1louA	d.58.14.1	10.723	6.438
1m8aA	d.9.1.1	4.847	2.703
1mhnA	b.34.9.1	4.582	3.124
1mszA	d.68.7.1	2.964	2.324
1mwzA	d.58.17.1	4.933	3.757
1n1jA	a.22.1.3	2.039	1.72
1n1jB	a.22.1.3	1.981	1.474
1ngrA	a.77.1.2	8.376	3.396
1nklA	a.64.1.1	3.052	2.075
1nkpA	a.38.1.1	1.566	1.923
1nkpB	a.38.1.1	1.681	2.435
1nlwA	a.38.1.1	2.656	2.075
1no8A	d.58.7.1	4.686	3.636
1npsA	b.11.1.1	10.589	6.07
1nr4A	d.9.1.1	6.509	1.756
1nu4A	d.58.7.1	8.815	5.69
1of9A	a.64.1.4	2.557	1.933
1ootA	b.34.2.1	5.962	4.281
1opcA	a.4.6.1	8.275	4.308
1oqpA	a.39.1.5	1.673	1.191
1or7C	a.180.1.1	2.61	2.091
1osdA	d.58.17.1	2.028	3.219
1ow5A	a.60.1.2	3.076	2.656
1p4wA	a.4.6.2	2.449	1.955
1p7iA	a.4.1.1	0.681	0.548
1pchA	d.94.1.1	11.721	3.417
1pfbA	b.34.13.2	5.849	4.26
1psrA	a.39.1.2	4.533	3.192
1ptfA	d.94.1.1	9.789	2.875
1pueE	a.4.5.21	9.242	3.437
1pufA	a.4.1.1	1.826	2.318
1pufB	a.4.1.1	0.927	1.571
1q08A	a.6.1.3	2.2	2.578
1q8lA	d.58.17.1	4.944	3.508

1qavA	b.36.1.1	8.28	2.868
1qjtA	a.39.1.6	2.404	4.061
1ufmA	a.4.5.47	2.735	3.605
1uhsA	a.4.1.1	2.482	2.191
1uhzA	d.50.1.1	3.575	2.645
1ulrA	d.58.10.1	10.143	5.837
1w0uA	a.4.1.4	2.45	1.973
1wfdA	a.7.14.1	1.826	1.582
1wgfA	a.21.1.1	3.311	2.882
1wgnA	a.5.2.1	2.834	2.185
1wh3A	d.15.1.1	9.776	4.224
1wm3A	d.15.1.1	1.76	2.743
1wriA	d.15.4.1	10.324	7.785
1xppA	d.74.3.2	11.26	4.904
2aw0A	d.58.17.1	3.48	2.487
2u2fA	d.58.7.1	7.298	4.461
3crdA	a.77.1.3	6.436	3.551
3ygsP	a.77.1.3	8.555	2.407
4ubpA	d.8.1.1	7.651	3.237
